# Cost-effectiveness analysis of insecticide-treated net distribution as part of the Togo Integrated Child Health Campaign

**DOI:** 10.1186/1475-2875-7-73

**Published:** 2008-04-29

**Authors:** Dirk H Mueller, Virginia Wiseman, Dankom Bakusa, Kodjo Morgah, Aboudou Daré, Potougnima Tchamdja

**Affiliations:** 1 Health Economics and Financing Programme, Department of Public Health and Policy, London School of Hygiene and Tropical Medicine, Keppel St., London WC1E 7HT, UK; 2 Division of Planning, Togolese Ministry of Health, Lomé, Republic of Togo; 3 National Malaria Control Programme, Togolese Ministry of Health, Lomé, Republic of Togo; 4 Division of Family Health, Togolese Ministry of Health, Lomé, Republic of Togo; 5 Director General, Togolese Ministry of Health, Lomé, Republic of Togo

## Abstract

**Background:**

To evaluate the cost-effectiveness of the first nationwide delivery of long-lasting insecticide-treated nets (LLITNs) as part of the 2004 measles vaccination campaign in Togo to all children between nine months and five years.

**Methods:**

An incremental approach was used to calculate the economic costs and effects from a provider perspective. Effectiveness was estimated in terms of malaria cases averted, deaths averted and Disability-Adjusted Life Years (DALYs) averted. Malaria cases were modelled using regional estimates. Programme and treatment costs were derived through reviews of financial records and interviews with key stakeholders. Uncertain variables were subjected to a univariate sensitivity analysis.

**Results:**

Assuming equal attribution of shared costs between the LLITN distribution and the measles vaccination, the net costs per LLITN distributed were 4.41 USD when saved treatment costs were taken into account. Assuming a constant utilization of LLITNs by the target group over three years, 1.2 million cases could be prevented at a net cost per case averted of 3.26 USD. The net costs were 635 USD per death averted and 16.39 USD per DALY averted, respectively.

**Conclusion:**

The costs per case, death and DALY averted are well within commonly agreed benchmarks set by other malaria prevention studies. Varying transmission levels are shown to have a significant impact on cost-effectiveness ratios. Results also suggest that substantial efficiency gains may be derived from the joint delivery of vaccination campaigns and malaria interventions.

## Background

Evidence on the effectiveness of insecticide-treated nets (ITNs) to prevent malaria in endemic regions is well established [1-5]. The cost-effectiveness of ITNs has also been widely reported particularly in the context of randomized control trials [6-8]. However, despite these positive results, mechanisms for public sector distribution of bed nets have struggled to match the coverage levels of vaccination campaigns [9]. The challenge remains in demonstrating the cost-effectiveness of delivering ITNs in non-trial settings at a regional or national level.

Recent studies indicate that vaccination campaigns have achieved high levels of coverage, especially if they are a one-off vaccination, such as the measles vaccine [10,11]. This has prompted campaigns, which distribute ITNs in combination with vaccinations, in an attempt to improve the coverage rates of ITNs, while minimizing any duplication of delivery costs across the two interventions. The results of trials at the sub-national level have been encouraging with household ownership of ITNs reaching levels of above 90% in Ghana and usage rates of 68% in Ghana and up to 77% in urban Zambia [12,13].

The Togo Integrated Child Health Campaign represents the first campaign on a national scale, in which various health interventions, including the distribution of a long-lasting insecticide-treated bed net (LLITN) and measles vaccination were jointly delivered to each household with at least one eligible child aged nine to 59 months [14-16]. Not all components of the campaign were delivered simultaneously; therefore this cost-effectiveness analysis (CEA) concentrates on the malaria and measles components of the campaign which were jointly implemented in December 2004. This study makes two important contributions to current knowledge in this area. This is the first economic evaluation of LLITN-distribution as part of an integrated health campaign. It provides an important opportunity to compare these results with other delivery mechanisms that have been applied at the national level such as the social marketing of bed nets [7]. Secondly, this study reveals that substantial costs are shared by the malaria and measles components of the campaign, highlighting the potential economies of scope inherent in the joint nature of the campaign.

## Methods

An incremental approach was used to estimate costs and effects. This involved comparing the campaign to a scenario of no public sector ITN distribution ('*do-nothing*' approach). Findings are presented from a provider perspective; only the costs and effects borne by the ministry of health and donors are considered. All relevant stakeholders were interviewed and asked to disclose their contribution to the campaign. Economic costs (reflecting full opportunity costs of resource use [17]) are divided into capital and recurrent costs and estimated using the ingredients approach in which all provider resources required in the delivery of the campaign are valued [18]. The main recurrent costs included personnel, overheads (such as office space, support staff, utilities, etc.) and transport. Overheads were apportioned according to the number and time of personnel dedicated to the campaign against total number of personnel. Capital costs (equipment, vehicles and buildings) were annualized over their estimated lifetime at a discount rate of 5% (Central Bank of West African States, BCEAO, personal communication). All other costs occurred during a period of less than one year and were therefore not discounted. Shared costs were apportioned equally between the malaria and measles components in the base case calculation. Costs were converted into US Dollars (USD) at the official exchange rate of 1 December 2004 when all major expenses were incurred (1 USD = 493 Franc CFA, Oanda Corporation).

Two sets of cost-effectiveness ratios were calculated. The first is based on gross estimates that do not take into account potential resource savings and the second set did (i.e. net cost-effectiveness ratios). Resource savings were derived by multiplying the average outpatient treatment costs for a child with malaria by the number of cases averted. This estimate was then adjusted for the percentage of children under the age of 5 that access a public health facility in the event of febrile illness in Togo (30% following a recent evaluation by the Malaria Control Programme of the Ministry of Health [19]).

To determine treatment costs, a facility survey was conducted in July 2005 in joint collaboration with the Togolese Ministry of Health (MoH). A total of 31 public, private-for-profit and private-not-for-profit facilities were surveyed in urban (20) and rural (11) areas and across all levels of care (three University Hospitals, seven Regional Hospitals, six District Hospitals and 15 peripheral health centres). Representatives from each facility were interviewed by other health care professionals (hired by the MOH) about staff time, equipment and consumables used to treat a typical malaria case as well as the overhead costs of the facility or department. Only outpatient costs were considered in this analysis in the absence of information on the proportion of inpatient vs. outpatient cases.

Health effects were measured in terms of cases of malaria averted, deaths averted and DALYs averted. The usage of LLITNs shortly after the campaign was 54% [20]. In the absence of information on LLITN utilization rates over time and on the duration of usage, a constant usage of LLITNs at 54% for three years by children under-five years of age was assumed. This assumption was adopted since the first two coverage surveys indicated that ownership of LLITNs had only marginally fallen nine months post-campaign [20]. Physical durability of LLITNs is dependent on the local context (number of washings, handling of nets), but recent experiences indicate that LLITNs of the kind used in Togo may last three years and longer (Gimnig, personal communication).

To calculate the number of deaths averted by the campaign, a reduction of all-cause mortality of 17% among one to 59 month-old children was applied as described in a Cochrane Review by Lengeler [3]. A figure of 182,000 live births per year in Togo as reported for the year 2004 [21] was used. From the overall under-five mortality of 140 per 1,000 live births per year the neonatal mortality rate (40/1,000 live births) was subtracted to obtain the mortality among one to 59-month old children [22-24]. Because the reduction in mortality described in the Cochrane Review was based on a higher utilization rate (70%), adjustments were made for the lower level of utilization in Togo. The impact of utilization rates on overall findings was tested in the sensitivity analysis.

In the absence of published morbidity and mortality data for children in Togo, recent estimates by the Child Health Epidemiology Reference Group (CHERG) were applied. These assume a median incidence of 1,209 malaria cases per year per 1,000 <5 children in middle Africa (including Togo) [25]. This was varied in the sensitivity analysis to a high transmission scenario reported from neighbouring Ghana (1,727 per 1,000) [26] and a low transmission scenario from neighbouring Benin (237 per 1,000) [27].

The number of DALYs averted was based on the estimated ratios of episodes, anaemia cases, neurological sequelae and deaths resulting from malaria cases as estimated by the CHERG [25]. Disability weights identified by the Global Burden of Disease Project were used; 0.211, 0.012 and 0.471 for malaria episodes, anaemia and neurological sequelae, respectively [28]. Life tables for the year 2000 for Togo indicated a life expectancy of 51.75 and 56.2 years for 0–1, and 1–5 year old children, respectively [29]. The duration of illness has been applied differently across studies [30]: in this study a duration of two weeks per malaria episode was assumed, eight weeks per anaemia case and an average of 35.4 years for treated and untreated neurological sequelae. DALYs were discounted at 3% without an age weighting correction as no consensus on its use has yet been demonstrated [30]. The impact of including an age weighting correction was tested in the sensitivity analysis.

It was also the intention of this study to examine the extent to which varying the attribution of shared costs between the malaria and measles components of the campaign influenced cost-effectiveness. In the main analysis, shared costs were attributed equally while in the sensitivity analysis all shared costs were allocated to measles *or *to malaria. Other uncertain variables subjected to a univariate sensitivity analysis included discount and exchange rates, lifetime of vehicles, price of LLITN, duration of LLITN usage, utilization rate of LLITNs, malaria transmission levels and the percentage of children with febrile illness accessing public health facilities. The study was part of a larger evaluation which gained ethical approval from the Togolese Ministry of Health and CDC, Atlanta.

## Results

Table 1 reveals that the gross economic cost of the Integrated Child Health Campaign (malaria and measles components) was 6.4 million USD. Capital expenditure (including the acquisition of LLITNs) accounted for 4.1 million USD and recurrent expenditure accounted for 2.3 million. More than half of total expenditure was attributed to the purchase of LLITNs (60.8%). Approximately 4.9 million USD, or 75.2% of total campaign costs, represents costs specific to the malaria component. Shared expenditure (i.e. expenditure that could not be attributed specifically to the malaria or measles component) accounted for 1.1 million USD or 16.4% of total costs. Shared costs mainly consist of salaries of personnel, their respective office space and overheads.

**Table 1:** Total campaign expenditure

**Line item category**	**total in USD**	**%**
*ITN*	3,917,325	60.7
Personnel	1,043,478	16.2
Consumables	565,025	8.8
Transport	433,176	6.7
Equipment	150,353	2.3
*Vaccines*	129,913	2.0
Travel	108,917	1.7
Room rental	110,895	1.7
Overheads	59,254	0.9
Fees (customs, bank charges etc.)	27,539	0.4
Services (handling, processing etc.)	13,908	0.2
Communication	9,325	0.1
Postage	451	0.0
Other	9,050	0.1
**TOTAL**	**6,448,695**	**100.0**

Table 2 summarizes the costs, effects and cost-effectiveness ratios for the intervention. Assuming that shared costs are split equally between the malaria and measles components (i.e. 50% each to the malaria and the measles component of the campaign), the gross costs of the malaria component alone were calculated at 5.38 million USD. With 1.2 million malaria cases averted over three years at a treatment cost per outpatient case of 3.79 USD, treatment costs averted amounted to 1.39 million USD. By subtracting these resource savings from total malaria programme costs, total net costs were 3.99 million USD for the malaria component of the campaign.

**Table 2:** Cost-effectiveness of the malaria component of the Togo Integrated Child Health Campaign

**Costs and savings**	
Total cost of malaria component	5.38 million USD
Total saved treatment costs	1.39 million USD
Net costs of the malaria component	3.99 million USD
**Effectiveness**	
Number of LLITNs distributed	904,500
Number of cases averted	1,223,862
Number of deaths averted	6,285
Number of DALYs averted	243,472
**Gross cost-effectiveness**	
Gross cost per LLITN distributed	5.95 USD
Gross cost per case averted	4.40 USD
Gross cost per death averted	856 USD
Gross cost per DALY averted	22.10 USD
**Net cost-effectiveness**	
Net cost per LLITN distributed	4.41 USD
Net cost per case averted	3.26 USD
Net cost per death averted	635 USD
Net Cost per DALY averted	16.39 USD

According to the MoH, a total of 907,500 LLITNs were distributed during the campaign [14]. This results in a gross cost of 5.95 USD and net cost of 4.41 USD per LLITN distributed, if shared costs are attributed equally.

It is estimated that 6,285 deaths and 1.2 million cases are averted between the ages of 9–59 months over a period of three years. The gross cost of the malaria component of the campaign equals 856 USD per death averted and 4.40 USD per case averted. The equivalent net costs were 635 USD per death averted and 3.26 USD per case averted.

Based on the assumption of constant usage of LLITN over three years, the total number of DALYs averted due to the malaria component of the campaign was 243,472. The largest share of malaria attributable DALYs is associated with discounted years of life lost (YLL) due to malaria; these amount to 189,249. The gross cost per DALY averted is calculated at 22.10 USD; the equivalent net cost is 16.39 USD.

Table 3 illustrates the results of the sensitivity analysis. One of the major assumptions underlying this evaluation concerns the attribution of shared costs: For the main analysis shared costs were attributed equally to the malaria and measles components of the campaign, which resulted in 16% of total programme costs allocated to measles and 83% to malaria. Attributing all shared costs to the malaria component (supposing that this component would have been implemented separately) would account for nearly 5.9 million USD for the malaria component or 92% of the total campaign expenditure. Alternatively, if all shared costs were attributed to measles, the revised malaria costs would be 1.6 million USD or 25% of total campaign costs. The gross and net cost per DALY averted would change from 22.10 and 16.39 USD to 24.28 and 18.56 USD, respectively, if all shared costs were absorbed by the malaria component or to 19.93 and 14.21 USD, respectively, if all shared costs were borne by the measles component.

**Table 3:** Sensitivity Analysis – Impact of varying major assumptions on gross and net costs per malaria case averted

**Variable**	**Change**	**Impact on gross cost per malaria case averted**	**Impact on net cost/savings per malaria case averted**
Attribution of shared campaign costs	All shared costs borne by measles component.	Decrease from 4.40 USD to 3.96 USD.	Decrease from 3.26 USD to 2.83 USD.
Attribution of shared campaign costs	All shared costs borne by malaria component.	Increase from 4.40 USD to 4.83 USD.	Increase from 3.26 USD to 3.69 USD.
Discount Rate	Decrease from 5% to 3%	Minimal decrease from 4.40 USD to 4.39 USD.	Minimal decrease from 3.26 USD to 3.25 USD.
Discount Rate	Increase from 5% to 7%	Minimal increase from 4.40 USD to 4.41 USD.	Minimal increase from 3.26 USD to 3.27 USD.
Lifetime of vehicles	From 8 and 10 years (depending on model) to 5 years	No change.	Minimal increase from 3.26 USD to 3.27 USD.
Exchange Rate	From 1/12/2004 to 1/10/2004	Minimal increase from 4.40 USD to 4.41 USD.	Minimal increase from 3.26 USD to 3.28 USD.
Exchange Rate	From 1/12/2004 to 1/1/2005	Minimal increase from 4.40 USD to 4.43 USD.	Minimal increase from 3.26 USD to 3.30 USD.
Price of LLITN	10% increase	Increase from 4.40 USD to 4.72 USD.	Increase from 3.26 USD to 3.58 USD.
	10% decrease	Decrease from 4.40 USD to 4.08 USD.	Decrease from 3.26 USD to 2.94 USD.
Duration of usage of LLITN	From 3 years to 2 years	Increase from 4.40 USD to 6.60 USD.	Increase from 3.26 USD to 5.46 USD.
	From 3 years to 4 years	Decrease from 4.40 USD to 3.30 USD.	Decrease from 3.26 USD to 2.16 USD.
Usage of LLITN among < 5 year old children	From current ratio of 54% in Togo to higher rates used in Cochrane review (70%)	Decrease from 4.40 USD to 3.39 USD.	Decrease from 3.26 USD to 2.25 USD.
Increase in transmission	From incidence of 1,209 per 1,000 p.a. to 1,727 per 1,000 p.a.	Decrease from 4.40 USD to 3.08 USD.	Decrease from 3.26 USD to 1.94 USD.
Decrease in transmission	From incidence of 1,209 per 1,000 p.a. to 237 per 1,000 p.a.	Increase from 4.40 USD to 22.43 USD.	Increase from 3.26 USD to 21.29 USD.
Percentage of febrile children accessing public health facilities	From 30% to 10%	No change by definition.	Increase from 3.26 USD to 4.02 USD.
	From 30% to 100%	No change by definition.	Decrease from 3.26 USD to 0.61 USD.

Changing the average duration of usage of an LLITN from three to two years increases the gross and net cost per DALY averted from 22.10 and 16.39 USD to 33.15 and 27.44 USD, respectively. The equivalent figures for four year usage are gross costs of 16.58 USD and net costs of 10.86 USD. Altering the usage for <5 year old children from 54% in Togo [20] to 70% as applied in the Cochrane review [3], results in decreases in gross cost per DALY from 22.10 to 20.72 USD and net cost per DALY from 16.39 to 13.76 USD.

The impact of changing incidence rates was also investigated by applying a high transmission scenario from Ghana (incidence of 1,727 per 1,000 per year [26]) and a low transmission scenario from Benin (incidence of 237 per 1,000 per year [27]). See Table 3 for the impact on the cost per case averted. Because these case studies did not describe the incidence of anaemia and neurological sequelae, the number of DALYs averted in these scenarios could not be calculated.

The number of children accessing public health facilities for the treatment of febrile illness varies significantly across sub-Saharan Africa. The current estimate for Togo is 30% [19]. Reducing this to 10% changes the net cost per DALY averted from 16.39 to 20.20 USD. An increase from 30% to 100% reduces the net cost per DALY averted from 16.39 to 3.05 USD.

Changing the discount rate, exchange rate, price of LLITN or lifetime of vehicles does not have a significant impact on campaign costs or cost-effectiveness ratios (Table 3). Including an age-weighting in the DALY calculations also had little impact with the gross and net costs per DALY averted changing from 22.10 and 16.39 USD to 22.17 and 16.44 USD, respectively.

## Discussion

This study estimates the cost-effectiveness of delivering LLITNs as part of a nationwide health campaign. In summary, we calculate gross and net costs of 4.40 and 3.26 USD per case of malaria averted; 856 and 635 USD per death averted; and 22.10 and 16.39 USD per DALY averted. The sensitivity analysis demonstrated that the results are robust to moderate changes in exchange and discount rates as well as changes in the price of LLITNs. Changes in the lifetime and/or usage rates of LLITNs as well as the rate of children with febrile illness accessing public health facilities had a greater influence over cost-effectiveness estimates.

Overall, the cost effectiveness of the campaign approach to deliver LLITNs in Togo compares favourably against other public sector ITN programmes. For example, in Ghana, the provision of ITNs and re-treatment was reported to cost 2,112 USD per death averted or 77 USD per DALY averted [31]. In Kenya, the cost per death averted through ITN distribution was estimated at 2,958 USD [32]. A modelling exercise undertaken by Goodman and colleagues reported costs of between 19 and 85 USD per DALY averted (1999 prices) for the distribution and re-treatment of ITNs across Sub-Saharan Africa [33,34]. More recently, the Disease Control Priorities Project (DCPP) found the cost per DALY averted fell between 11 and 17 USD depending on the frequency of re-treatment and insecticide used for re-treatment of conventional ITNs [35].

The Togo results can also be compared against other methods for distributing bed nets. For example, a recent cost-effectiveness analysis (2003) of social marketing of ITNs in two rural districts of Tanzania estimated that it cost 1,560 USD per death averted, 57 USD per DALY averted and 8.30 USD per net distributed [36]. Perhaps more relevant are the results from one of the few social marketing studies of LLITNs conducted at the national level. The study from Malawi (2005) which distributed almost 1.5 million conventional ITNs, estimated that it cost 2.49 USD to distribute each net [7] compared to 5.95 USD under the Togo study where approximately 907,000 nets were distributed. Further comparisons can be made between this study and other malaria control mechanisms such as indoor-residual spraying (IRS) which has been estimated to cost between 16 and 58 USD per DALY averted [32,33] and most recently, between 5 and 34 USD [35]. Chemoprophylaxis for children can cost anywhere between 3 and 41 USD per DALY averted and intermittent presumptive treatment of pregnant mothers (IPTp) between 4 and 29 USD per DALY averted [32,33]. Estimates from this study lie within the cost per DALY range for both IRS and IPT. The results from this study also fall below the benchmark of 30 USD per DALY for highly cost-effective interventions which was recommended by the World Health Organization in 1996 [37].

While it can be useful to contextualize cost-effectiveness estimates in this way, there are some important differences across studies that can undermine comparability. For example, most existing studies focus on ITNs (with the need for periodic re-treatment) as opposed to LLITNs. There is also considerable variation in the outcome measures used and the rates of transmission applied. Some studies acknowledge resource savings while others do not. 'Benchmarks' and 'thresholds' are only a guide to decision-makers. With most African governments currently devoting less than 5 USD per person annually to public health [38] it is difficult to see how a publicly funded bed net programme could be sustained even if it represented good value for money as is the case with delivering LLITNs as part of the Togo health campaign.

A number of methodological issues must also be borne in mind when interpreting the results of this analysis. Firstly, in the absence of pre- and post-campaign incidence data on malaria, all cost-effectiveness estimates are based on regional averages. While there is no particular reason to think that Togo is an outlier in this respect, this can only be confirmed through the collection of primary effectiveness data. Secondly, estimates are based on the assumption that utilization will be guaranteed at the current rate (or above) for no less than three years. This assumption needs to be tested. Thirdly, it is conceivable that additional resources may be required to maintain current coverage rates of LLITNs and this would increase the overall cost of this intervention. Fourthly, in the absence of data on the percentage of hospitalizations attributable to malaria, resource savings are based on the average cost of outpatient treatment only. Net costs for Togo are, therefore, likely to be conservative since they do not include the relatively high costs of inpatient care. Finally, cost-effectiveness was assessed from a provider viewpoint. Those wishing to explore the social desirability of this type of intervention should consider any additional costs and benefits to recipients of LLITNs and their families. For example, the estimates of effectiveness presented here do not acknowledge the possible effects of LLITNs on family members sleeping in the same room as an LLITN user. Savings in treatment costs to households using LLITNs have also been excluded from this analysis. It is expected that the inclusion of such benefits will further promote the cost-effectiveness of LLITN distribution through mass health campaigns.

## Conclusion

On the basis of cost-effectiveness, the results of this analysis suggest that delivering LLITNs as part of a wider health campaign compares well with other malaria control interventions and results are even more favourable if averted treatment costs are considered. These findings also suggest that there are potential efficiency gains to be had by incorporating LLITNs into health campaigns like that of Togo. The total cost of the Togo campaign was 6.5 million USD. Of this amount, 1.1 million USD represents shared costs between the two components of the campaign. Assuming equal attribution of shared costs, the gross cost per LLITN distributed was calculated at 5.95 USD. The sensitivity analysis revealed that attributing all shared costs to the malaria component increases the distribution costs per LLITN from 1.62 USD to 2.21 USD. While the true allocation of shared costs can only be determined through separate costings of the measles and malaria components of the campaign, this analysis implies that reductions in average total costs may be achieved by extending the range of goods delivered in this type of campaign to include LLITNs.

## Authors' contributions

DM and VW conceptualized the overall cost-effectiveness study. DM and DB planned and supervised the data collection both on the campaign expenditure and the treatment cost survey. DM, DB, KM and AD finalized the questionnaires for the treatment cost survey and supervised the data collection. PT advised on the methods for the treatment cost survey and on the general cost-effectiveness study within Togo. DM designed the cost-effectiveness calculation and sensitivity analysis and prepared the manuscript, which was reviewed and discussed with co-authors. VW sourced funding for the project, advised and contributed to the cost-effectiveness calculations and contributed significantly to the final draft of the manuscript. All authors read and agree to the final version of this manuscript.
